# Online experimental research on the psychological capital development of new venture entrepreneur under the COVID-19 pneumonia epidemic

**DOI:** 10.3389/fpsyg.2022.963439

**Published:** 2023-01-11

**Authors:** Na Zeng, Ming Zhang, Shanna Fu, Qin Xiao, Tahira Javed

**Affiliations:** ^1^College of Economics and Management, China Three Gorges University, Yichang, China; ^2^School of Business and Management, Shanghai International Studies University, Shanghai, China

**Keywords:** COVID-19, new venture entrepreneur, psychological capital development, structured reading material, online experiment, mindfulness intervention

## Abstract

The global economy has been severely traumatized by the COVID-19 pandemic, and new ventures are under tremendous pressure to survive. This paper explores whether structured reading materials and mindfulness intervention can develop entrepreneurs’ psychological capital and whether there are different effects on different development methods. We recruited 112 new venture entrepreneurs and carried out an online experiment using the experimental group, the control group, and the pre-test and post-test design; at last, 83 of whom participated in the process can be assessed for psychological capital development effect. The research findings indicated that structured reading materials could effectively improve the overall psychological capital of the subjects (*t* = −5.574，*p* < 0.01) and impact in every dimension, including self-efficacy (*Z* = −2.858, *p* < 0.01), hope (*t* = −3.560, *p* < 0.01), resilience (*t* = −4.368, *p* < 0.01) and optimism (*Z* = −3.300, *p* < 0.01). In comparison, mindfulness intervention can improve the subjects’ overall psychological capital (*Z* = −3.293, *p* < 0.01) and improve levels of self-efficacy (*Z* = −2.285, *p* < 0.01), hope (*t* = −4.244, *p* < 0.01), resilience (*t* = −3.167, *p* < 0.01), but limited effect on the optimism (*t* = −1.955, *p* > 0.05); In addition, there is no significant difference between structured reading materials and mindfulness intervention according to the statistical analysis.

## 1. Introduction

COVID-19 is a specific event at the time of VUCA (Volatility, Uncertainty, Complexity, Ambiguity), plaguing the world (Bennett and Lemoine, 2014). It continues to rage even though the government has taken several positive measures worldwide. People in daily life face challenges (Dirzyte et al., 2022) because the significant impact of the COVID-19 pandemic on global society has inflicted mental health problems across the world. Psychological morbidities such as stress, anxiety, insomnia, and depression have been reported not only by COVID-19 patients and healthcare workers but also by the general population (Krishnamoorthy et al., 2020). How to effectively cure people’s psychological trauma as a result of the epidemic’s effects is an essential task for medical workers, psychologists, and government agencies.

As a specific group, new venture entrepreneurs also suffer from body and mind problems. They not only have to face the challenges of newness that are unique to entrepreneurship (Stinchcombe, 1965), but they also have to bear the risks brought by the uncertain environment and falling into a deep financial crisis under the impact of COVID-19 (Zhang, 2020). The International Trade Centre (ITC) 2020 survey of 4,467 companies in 132 countries found that almost 67% of micro-and small businesses worldwide have been affected significantly by the pandemic and were likely to shut down their operations (SME Competitiveness Outlook, 2020). The research reveals that entrepreneurs still remain fairly pessimist, even though COVID-19 has gradually faded out of people’s talks or the society is stepping into a post-epidemic era (Li et al., 2020). For start-up business, the entrepreneur is the firm, and the firm is the entrepreneur (Lewis and Churchill, 1983); all kinds of entrepreneur capital constitute the most critical asset of a new venture (Erikson, 2002). Studies illustrate that the founders’ ability to cope with stress can be a critical factor in determining business sustainability (Davis-Brown and Salamon, 1987), in which psychological capital is significant. Hmieleski and Carr (Hmieleski and Carr, 2008) confirm that, for entrepreneurial performance, the explanatory power of psychological capital exceeds that of human capital and social capital. Therefore, it is significant to help entrepreneurs escape the dilemma caused by entrepreneurial activities and COVID-19.

The psychological pressure of new venture entrepreneurs can be alleviated in various ways. Firstly, from the macro environment, protecting the property safety of new venture entrepreneurs and providing financial support and tax relief can release their pressure; Secondly, entrepreneurs’ hope, resilience, optimism, and self-efficacy can be improved through psychological capital development (Zhang et al., 2012). Studies on non-new venture entrepreneurs show that psychological capital is a positive psychological factor (Zhou et al., 2021), and psychological capital development can bring positive psychological changes (Da et al., 2020), promote positive behavioral responses (Stratman and Youssef-Morgan, 2019), and improve the performance of individuals and organizations (Carter and Youssef-Morgan, 2019). However, existing research on psychological capital development mainly focuses on groups easier to gather, such as students and corporate employees. In contrast, the research subjects who are not easy to assemble or unwilling to accept external intervention in developing psychological capital are rare, such as entrepreneurs. At the same time, entrepreneurs are quite independent and inaccessible because of time and location limits. So the traditional PCI (Psychological Capital Intervention) model may not be suitable for them. Research showed that psychological interventions should be integral to future crisis management plans (Duan and Zhu, 2020; Rutkowska et al., 2022).

This research recruited new venture entrepreneurs (founders or current leaders of new ventures that have not been established for over 6 years) in Hubei Province as the research objects. We built a series of flexible and sustainable psychological capital development programs for research. The program was implemented with online experiments, including structured materials reading and mindfulness intervention, then evaluated the effects among the experimental and control groups by pre-test and post-test. We aimed to explore the effect differences among different interventions and provide inspiration for the psychological capital development of new venture entrepreneurs in stressful situations.

## 2. Literature review and research hypothesis

Psychological capital is a positive psychological state that individuals show in growth and development (Luthans and Youssef, 2004). Regarding the composition of psychological capital, the four-dimensional theory is currently the mainstream (Avey et al., 2010; Jafri, 2012): self-efficacy, hope, resilience, and optimism. Many studies take psychological capital as antecedent variables and mediators using correlational coefficient, structural equation model, et al. and proved its importance (Pompuang et al., 2019; Suksod et al., 2019). Focusing on the field of entrepreneurship, the four major constructs can be explained as how they facilitate the entrepreneurial journey. Self-efficacy can be referred to as having the confidence to achieve a particular goal, enabling an individual to continue overcoming all possible challenges. Hope signifies the positive energy that motivates an individual to explore potential opportunities and win competitions. Resilience enables a person to control their emotional stability, which could help to overcome challenges in difficult situations. Optimism empowers an individual to expect a positive outcome for the processes and bring greater success for the firm (Tang, 2020).

**Figure 1:**
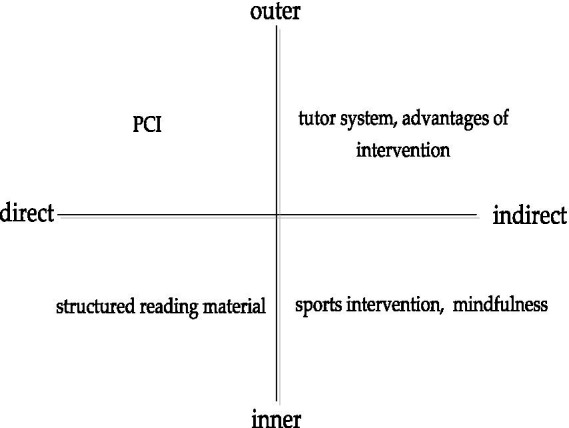
Psychological capital development methods based on typological framework.

Psychological capital, like human capital and social capital, could be managed and invested; unlike traditional capital and tangible assets, it is achieved even with little investment (Luthans et al., 2007). Psychological capital development could enable individuals to obtain competitive advantages in human resources and ultimately achieve sustained and real growth in work performance and organizational performance (Luthans et al., 2018). Baron et al. found that entrepreneurs with higher psychological capital have higher welfare because psychological capital can form good entrepreneurial competencies (Baron et al., 2016). Baluku et al. compared the predictive power of psychological and venture capital on entrepreneurial success. It was found that venture capital, which includes knowledge and practical experience, could only explain 8.5% of the variation in entrepreneurial success, while psychological capital could explain as much as 34% (Baluku et al., 2016). Many interventions are used in psychological capital development, such as the tutor system, advantages of intervention, structured reading material, PCI model, sports intervention, and mindfulness. Based on typology strategy (Miller, 1996), this study combines existing development methods according to outer and inner, direct and indirect dimensions, and highly summarized four types (see Figure 1).

Different types of psychological capital development have been tested in experimental research and have their advantages. Inner psychological capital development with low cost can be independently developed without intervention and is not limited by time and place. Therefore, it can be implemented on a large scale in different populations. Outer psychological capital development is challenging because it needs experts’ external intervention and the development objects’ cooperation and response. However, it is more maneuverable, and the intervention effect is more guaranteed. Direct psychological capital development is mainly based on the PCI model, and the intervention effect for the four dimensions of psychological capital is relatively guaranteed. However, it excludes the potential dimension of psychological capital, so the limitations are apparent. Indirect psychological capital development adopts the strategy of references or transplantations. It is economical and straightforward, but its effect still needs to be tested. Whether outer, inner, direct, or indirect, psychological capital development has its strength and weakness. The best intervention method is to realize the complementarity of strength under specific situations.

Entrepreneurs with high self-efficacy may be unwilling to accept outer therapeutic intervention. The inner psychological capital development method can reduce the participants’ resistance and improve the intervention effect. At the same time, the inter-psychological capital development method reduces the intervention of other objects in the intervention process. So, the problems that exist in the online intervention can be solved, such as weak interaction. This paper focuses on the inner psychological capital development methods by recruiting entrepreneurs as research subjects and using experiment methodology. In addition, the study selects structured reading materials and mindfulness intervention from the inner-direct and inner-indirect development methods, respectively, to explore whether there are differences between the direct and indirect psychological capital development methods.

### 2.1. Structured reading material

Structured reading materials belong to Bibliotherapy, an applied science involving genetics and psychology (Wang and Xu, 2021). The concept of “Bibliotherapy” was first proposed by Crothers (1917), and it was not introduced into China until the end of the 20th century (Zhu and Zhang, 2012). Bibliotherapy takes literature as the carrier and uses the principles of resonance, purification, balance, suggestion, and comprehension to promote the psychological development of readers to achieve the goal of treating mental illness or maintaining mental health (Wang and Fu, 2003). Although there are few direct studies on Bibliotherapy and psychological capital, its effect on improving the dimensions of psychological capital provides indirect evidence of the effectiveness of structured reading materials. For example, Feld had written on the inspirational role of the stories of successful local entrepreneurs whose narratives detail how their start-ups achieved international success (Feld, 2020). Zhang et al. (2014) use structured reading materials to develop the psychological capital of Chinese enterprise employees; the result shows that the psychological capital and work performance of employees have increased significantly, and the development effect on the overall psychological capital, hope dimension, and work performance last for 3 months. Markowska and Lopez-Vega (2018) have identified stories act as functional tools for individuals, particularly entrepreneurs’. Hence, we hypothesize that:

*Hypothesis 1 (H1)*: Structured reading materials could improve the psychological capital of new venture entrepreneurs.

### 2.2. Mindfulness intervention

The Eastern perspective of mindfulness is based on the Buddhism thought tradition that emphasizes attention to and awareness of present moments (Weick and Putnam, 2006). It can avoid immersing in past or negative emotional experiences, maintain an open and accepting attitude, improve emotional regulation, and help individuals work and live with a more positive attitude (Good et al., 2016; Zheng and Ni, 2018). Several mechanisms have been promoted in mindfulness exercises, including decentering, facilitating the separation of one’s ego from negative situations or events (Feldman et al., 2010). This is especially important for entrepreneurs, who often face significant obstacles and setbacks when starting a company. Charoensukmongkol (2019) revealed that in times of crisis, individual mindfulness is positively associated with entrepreneurs’ improvisational behavior and business performance.

Although there are few mindfulness intervention studies about entrepreneurs, they all support the effectiveness of mindfulness intervention in psychological capital development. For example, a study conducts Mindfulness Based Stress Reduction (MBSR) on adults of different ages and showed that 8 weeks of MBSR significantly improved the four dimensions of psychological capital (Jain and Singh, 2016). One randomized control study finds that a one-week workplace support group intervention reduced depression symptoms among workers (Ahola et al., 2012). Sparks and Ring (2022) demonstrated that 6-week rowing-specific mindfulness intervention could encourage mindfulness and aid performance. One meta-analysis provides evidence that mindfulness-based programs effectively promote employees’ health and well-being in various occupational settings (Vonderlin et al., 2020). Yet, a systematic review found that mindfulness-based programs do not affect every nonclinical setting (Galante et al., 2021). Hence, we hypothesize that:

*Hypothesis 2 (H2)*: Mindfulness intervention could improve the psychological capital of new venture entrepreneurs.

### 2.3. Effect comparison

Structured reading materials and mindfulness interventions are both inner development methods and can be independently developed without the guidance of outer experts. The difference is that the structured reading materials focus on four dimensions of psychological capital, respectively, while the mindfulness intervention targets the whole psychological capital. In fact, due to the open nature of psychological capital, in the process of intervention, the effect of improvement may be scattered on the existing dimensions and the potential dimensions of psychological capital that have not been included. From this perspective, the effectiveness of mindfulness interventions and structured reading materials is unknown. However, from the perspective of measurement scales, the PCQ-12 (Psychological Capital Questionnaire -Short Version) selected in this paper is a combination of subscales based on self-efficacy, hope, resilience, and optimism, which can effectively capture the four dimensions of improvement after the intervention, and structured reading materials target the questionnaire. But for mindfulness intervention, the scale’s validity in the potential dimension of psychological capital may be insufficient. As a result, different intervention effects may occur.

At the same time, the high-quality development of the private economy has been seriously threatened by COVID-19. New venture entrepreneurs face difficulties in their operation and development and have to bear the impact of the outer environment. Under tremendous pressure to survive, the brain is in an overactive action mode. During the experimental intervention, the subjects were required to activate the existence mode and stop the brain from overthinking. Structured reading materials represent passive acceptance, and mindfulness intervention requires more active participation. Hence, we hypothesize that:

*Hypothesis 3 (H3)*: The effect of structured reading materials on developing new venture entrepreneurs’ psychological capital is better than mindfulness intervention.

## 3. Research design

### 3.1. Participants

In this study, G* Power 3.1.9.7 software (Faul et al., 2007) was used to calculate the sample size required. The effect size was set as 0.4, and α was set as 0.05 (Luthans et al., 2010). Under the premise of statistical power 1–*β* = 0.8, it was found that a minimum of 66 objects were required for one-way ANOVA.

We recruited some objects from various business incubators in Hubei Province to ensure that all experimental subjects were new venture entrepreneurs. The other part was recruited in a snowball effect through an acquaintance of entrepreneurs. A total of 112 participants were recruited. The recruited participants were randomly assigned to balance the experimental and control groups’ demographic characteristics. The questionnaires were distributed and collected online. One hundred and eight questionnaires were collected in the pre-test. Post-recognition methods (response pattern recognition and response time recognition) were used to control the unconscientious respondents in the survey, and some questionnaires with missed answers or obvious response tendencies were deleted (Zhong et al., 2021). In addition, according to the question of “enterprise establishment years,” the part of subjects whose establishment years were more than 6 years were excluded. According to the above criteria, 93 valid questionnaires were collected in the pre-test, and 83 valuable questionnaires in the post-test, including 28 in the structured reading materials group, 27 in the mindfulness intervention group, and 28 in the control group. The demographic variables of all participants in the post-test are shown in Table 1.

**Table 1:** Descriptive characteristics of the sample.

Statistical category	Sample characteristics	Structured reading materials group		Mindfulness intervention group		Control group	
		Frequency	Percent (%)	Frequency	Percent (%)	Frequency	Percent (%)
Gender	Males	16	57.14	14	51.85	15	53.57
	Females	12	42.86	13	48.15	13	46.43
Age	Under 30	22	78.57	18	66.67	22	78.57
	30–45	4	14.29	6	22.22	4	14.29
	Above 45	2	7.14	3	11.11	2	7.14
Education	High school or below	7	25	8	29.63	8	28.57
	College	8	28.57	9	33.33	8	28.57
	Undergraduate	8	28.57	6	22.22	9	32.14
	Postgraduate	5	17.86	4	14.82	3	10.72
Enterprise type	State-owned and state-owned holding	1	3.57	2	7.41	4	14.29
	State-owned enterprise	2	7.14	0	0	1	3.57
	Private enterprise	21	75	21	77.78	19	67.86
	Foreign-funded enterprises	0	0	0	0	1	3.57
	Others	4	14.29	4	14.81	3	10.71
Industry	Manufacturing	1	3.57	1	3.7	4	14.29
	Wholesale and retail	7	25	5	18.52	6	21.43
	Leasing and business services	2	7.14	1	3.7	1	3.57
	Real estate	1	3.57	1	3.7	0	0
	Finance	2	7.14	0	0	1	3.57
	Accommodation catering industry	9	32.15	12	44.45	7	25
	Information technology and hardware and software industry	2	7.14	0	0	1	3.57
	Others	4	14.29	7	25.93	8	28.57
Number of employees	Under 20	20	71.43	15	55.56	20	71.43
	21–50	6	21.43	6	22.22	3	10.71
	51–100	1	3.57	3	11.11	1	3.57
	Above 100	1	3.57	3	11.11	4	14.29

Only the demographic information of 83 objects with valuable questionnaires.

### 3.2. Experimental design

“Online Experiment” is a research method based on the internet that follows experimental research criteria to test the causal relationship between variables (Chen and Konstan, 2015). Compared with traditional offline experiments, online experiments have natural advantages in expanding the size and type of objects and reducing research costs (Weng and Li, 2020). New venture entrepreneurs are highly independent, have insufficient time, and are difficult to gather (Zhang et al., 2012). Meanwhile, under the COVID-19 epidemic, online experiments have created conditions for research free from time and place limitations.

Considering entrepreneurs’ tight schedules, this study compressed the intervention time to 30–50 min and completed the whole experiment by taking short-term intervention and post-training assignments.

The structured reading material mainly referred to the relevant contents (Li, 2012; Wang, 2012), including two parts: understanding the connotation of psychological capital and its four dimensions and analyzing the effect of psychological capital. Psychological capital promotion strategies were presented to the participant subject, and materials were introduced from each dimension based on the PCI model. In addition to the 30-min PDF materials, the intervention process was supplemented by reading “Psychological Capital: Developing the Human Competitive Edge” (Luthans et al., 2008) and “Psychological Capital and Beyond” (Luthans et al., 2018). Moreover, PDF materials and electronic books were distributed to the online experimental group.

According to previous studies, this study included two parts: mindfulness introduction materials and mindfulness practice. Because of the low popularity of mindfulness intervention, the introductory material of this study summarized the basic content of mindfulness, including the connotation and key points of mindfulness intervention, the positive impact of a mindfulness intervention on individuals and enterprises, the mechanism of a mindfulness intervention, operational essentials of several common mindfulness interventions. Mindfulness exercises involved breathing and body scanning awareness and were trained primarily by subjects following audio material. In addition to PDF import materials and audio files of mindfulness training, the study recommended WeChat public accounts related to mindfulness training and mobile phone software specialized for mindfulness training.

Participants were asked to complete structured reading materials and mindfulness intervention content for about 30 min for 1 week. During this period, the participants were reminded daily in the group. They had an interactive exchange of questions and thoughts about the intervention, while the study excluded subjects who did not participate in training by distributing a post-test questionnaire. The specific development plans of structured reading materials and mindfulness invention are as Supplementary

### 3.3. Questionnaire mental procedure

The study was officially recruited at the beginning of October 2020, and eligible subjects were invited to join the online experimental QQ group voluntarily. The informed letter was distributed after we recruited sufficient participants, and a single-blind experimental design was adopted. After the experiment, the purpose of the experiment was explained.

This study was conducted with one short-term intervention and two questionnaire surveys. The phone number or email account matched the two tests in each survey. The first questionnaire was a pre-test, and the researchers conducted online testing. Demographic information and psychological capital level data of objects were obtained through the Wenjuanxing. After the pre-test, we numbered all the subjects’ nicknames in the group, and all the subjects were divided into three groups by the SPSS random number generator. Then subjects were required to enter the corresponding QQ group, respectively. During this time, the control group received no experimental intervention. One week after the intervention, questionnaires were issued to the subjects for the post-test. To effectively control the learning effect, this study rearranged the question order of the scale in the post-test. After the experiment, the researcher shared the intervention materials with all the subjects and paid the labor fee.

### 3.4. Variable measurement

In this study, the PCQ-12 contained 12 questions (Avey et al., 2011), which were divided into four dimensions: self-efficacy (3 questions), hope (4 questions), resilience (3 questions), and optimism (2 questions). To avoid central tendency, this study used a Likert 6-point scale to ask objects to evaluate their attitudes toward 12 questions, with 1 representing “strongly disagree” and 6 representing “strongly agree.” The higher the score, the higher the level of psychological capital.

## 4. Experimental results and analysis

### 4.1. Reliability and validity analysis

#### 4.1.1. Reliability analysis

The Cronbach’s α coefficient of the total psychological capital table was 0.873, and the Cronbach’s α coefficient of each dimension measurement item was above 0.7, indicating a good reliability coefficient. The most appropriate statistic is the Spearman–Brown coefficient when reporting the reliability of the two-item scale (Eisinga et al., 2013). Spearman–Brown split-half reliability was 0.765, and Guttman split-half reliability was 0.765 for the optimism dimension subscale. Analysis suggested that the scale is suitable for this study’s measuring tool.

#### 4.1.2. Validity analysis

AMOS 22.0 was used in this study to conduct confirmatory factor analysis on the four-factor structure of the psychological capital questionnaire and to judge the construct validity according to the fitting index in the structural equation model (Zhang and Wang, 2018). Results showed that *χ*^2^/df < 2,0.9 < IFI < 1,0.9 < CFI < 1,0 < NFI < 1,0 < RMSEA <0.1. *χ*^2^/df, IFI, and CFI were ideal, while NFI and RMSEA were not optimal but within an acceptable range. The model-fitting result met the standard, indicating that the questionnaire could fit the data well.

### 4.2. Comparison of pre-test scores between groups

To observe whether there were significant differences in pre-test scores between the structured reading materials group, one-way ANOVA and nonparametric tests were performed in the mindfulness intervention group and the control group. If the data met normal distribution and homogeneity of variance, a one-way ANOVA was adopted; otherwise, the nonparametric test was assumed (Lan, 2011).

Considering the small sample size (83 < 100), this study conducted a normal distribution test on the pre-test psychological capital of the experimental group and the control group using the Shapiro–Wilk method. The results did not obey the normal distribution hypothesis.

In this study, the Kruskal-Wallis method of the nonparametric test was further used to test the homogeneity of psychological capital between the experimental and control groups. The data of each group showed skewed distribution and was represented by median (quartile), i.e., *p*_50_ (*p*_25_–*p*_75_), as shown in Table 2.

**Table 2:** Pre-test in experimental group and control group based on Kruskal–Wallis method.

Variable	Structured reading materials group	Mindfulness intervention group	Control group	*χ* ^2^	*p*
Psychological capital	4.708 (4.417–4.958)	4.833 (4.500–5.000)	4.583 (4.271–5.000)	1.759	0.415
Self-efficacy	5.000 (4.667–5.000)	5.000 (4.667–5.333)	5.000 (4.667–5.250)	1.107	0.575
Hope	4.625 (4.250–5.000)	4.750 (4.250–5.000)	4.250 (4.063–4.750)	3.191	0.203
Resilience	4.500 (4.000–5.000)	4.667 (4.333–5.000)	4.500 (3.750–5.000)	1.088	0.580
Optimism	5.000 (4.417–5.000)	5.000 (4.500–5.500)	5.000 (4.000–5.000)	1.816	0.403

As shown in Table 2, there were no significant differences in the total score of psychological capital (*H* = 1.759, *p* = 0.415), self-efficacy (*H* = 1.107, *p* = 0.575), hope (*H* = 3.191, *p* = 0.203), resilience (*H* = 1.088, *p* = 0.580) and optimism (*H* = 1.816, *p* = 0.403). The results showed that the three groups were homogeneous and comparable. The initial psychological capital of each group was at the same baseline level, which provided a premise for the effectiveness of the subsequent intervention.

### 4.3. Comparison of pre and post-measurements within groups

The Shapiro–Wilk test showed that the difference values between the pre-test and post-test of psychological capital, hope, and resilience were normally distributed in the structured reading materials group (*p* > 0.05), the difference values of hope, resilience, and optimism were normally distributed in the mindfulness intervention group (*p* > 0.05). Only the difference values of psychological capital were normally distributed in the control group (*p* > 0.05). According to the normal distribution test results, matched sample T-test was performed on the variables to the normal distribution (see Table 3), and the Wilcoxon test was used on the remaining variables (see Table 4).

**Table 3:** Matched sample *t*-test.

Group	Variable	Mean		Standard deviation	*t*	Sig
		Before intervention	After intervention			
Structured reading materials group	Psychological capital	4.696	5.116	0.398	−5.574	0.000
	Hope	4.634	5.054	0.624	−3.560	0.001
	Resilience	4.560	5.036	0.577	−4.368	0.000
Mindfulness intervention group	Hope	4.509	5.056	0.669	−4.244	0.000
	Resilience	4.679	5.111	0.709	−3.167	0.004
	Optimism	4.852	5.130	0.738	−1.955	0.061
Control group	Psychological capital	4.601	4.595	0.430	0.073	0.942

**Table 4:** Wilcoxon test (P_25_, P_50_, P_75_).

Group	Variable	Before intervention	After intervention	*Z*	*p*
Structured reading materials group	Self-efficacy	5.000 (4.667–5.000)	5.000 (4.667–5.667)	−2.858	0.004
	Optimism	5.000 (4.125–5.000)	5.250 (4.500–5.875)	−3.300	0.001
Mindfulness intervention group	Psychological capital	4.833 (4.500–5.000)	5.000 (4.917–5.500)	−3.293	0.001
	Self-efficacy	5.000 (4.667–5.333)	5.000 (5.000–5.667)	−2.285	0.022
Control group	Self-efficacy	5.000 (4.667–5.250)	5.000 (4.083–5.000)	−1.660	0.097
	Hope	4.250 (4.063–4.750)	4.500 (3.813–4.750)	−0.133	0.894
	Resilience	4.500 (3.750–5.000)	4.667 (4.000–5.000)	−0.170	0.865
	Optimism	5.000 (4.000–5.000)	4.500 (4.000–5.500)	−0.371	0.711

*P*_25_, *P*_50_, and *P*_75_ represent the lower quartile, median and upper quartile in the interquartile range.

Combined with Tables 3 and 4, the pre-test and post-test differences in psychological capital and all dimensions in the structured reading materials group were significant at 0.01. Specifically, in terms of psychological capital, *t* = −5.574, *p* = 0.000; Self-efficacy, *Z* = −2.858, *p* = 0.004; Hope, *t* = −3.560, *p* = 0.001; Resilience, *t* = −4.368, *p* = 0.000; Optimism, *Z* = −3.300, *p* = 0.001. Therefore, before and after the intervention of structured reading materials, the scores of psychological capital and the four dimensions were significantly different, and the scores in the post-test were higher than those in the pre-test. That is, structured reading materials could significantly improve the overall psychological capital and the four dimensions individually. H1 was supported.

There were statistically significant differences between the pre-test and post-test scores of psychological capital, self-efficacy, hope, and resilience in the mindfulness intervention group, while there were no statistically significant differences between the pre-test and post-test means of optimism. Specifically, in terms of psychological capital, *Z* = −3.293, *p* = 0.001; Self-efficacy, *Z* = −2.285, *p* = 0.022; Hope, *t* = −4.244, *p* = 0.000; Resilience, *t* = −3.167, *p* = 0.004; Optimism, *t* = −1.955, *p* = 0.061. Therefore, the scores of psychological capital, self-efficacy, hope, and resilience were significantly different before and after the mindfulness intervention. Although the score of the optimism dimension improved, it did not reach the level of significant difference. Overall, mindfulness intervention significantly improved participants’ psychological capital, self-efficacy, hope, and resilience, but had a limited effect on optimism. H2 was partially supported.

There were no statistically significant differences of the control group’s psychological capital between the pre-test and post-test results in the four dimensions. Specifically, in terms of psychological capital, *t* = 0.073, *p* = 0.942; Self-efficacy, *Z* = −1.660, *p* = 0.097; Hope, *Z* = −0.133, *p* = 0.894; Resilience, *Z* = −0.170, *p* = 0.865; Optimism, *Z* = −0.371, *p* = 0.711.

### 4.4. Comparison of post-test scores between groups

According to the Shapiro–Wilk test, the post-test data in the dimensions of psychological capital and hope obeyed the normal distribution (*p* > 0.05), while the significance in other dimensions was not completely higher than 0.05, which did not obey the normal distribution hypothesis. Whether the psychological capital and hope dimensions were consistent with the homogeneity of variance hypothesis was further tested (see Table 5).

**Table 5:** Homogeneity of variance test.

Variable	Levene statistics	df1	df2	Sig
Psychological capital	1.145	2	80	0.323
Hope	0.401	2	80	0.671

As shown in Table 5, *p* values were all higher than 0.05, and the homogeneity of variance hypothesis of psychological capital and hope dimension was established. To compare the effects of different intervention methods, this study conducted a one-way ANOVA on psychological capital and hope dimensions that met the assumption of normal distribution and homogeneity of variance. The nonparametric test used the Kruskal–Wallis test for other variables (see Tables 6 and 7).

**Table 6:** One-Way ANOVA $\left(\bar{x}\pm s\right)$ .

Variable	Structured reading materials group	Mindfulness intervention group	Control group	*F*	*P*
Psychological capital	5.116 ± 0.493	5.105 ± 0.503	4.595 ± 0.602	8.607	0.000
Hope	5.054 ± 0.558	5.056 ± 0.530	4.438 ± 0.651	10.403	0.000

**Table 7:** Kruskal–Wallis test (*P*_25_, *P*_50_, *P*_75_).

Variable	Structured reading materials group	Mindfulness intervention group	Control group	*χ* ^2^	*P*
Self-efficacy	5.000 (4.667–5.667)	5.000 (5.000–5.667)	5.000 (4.083–5.000)	6.377	0.041
Resilience	5.000 (4.667–5.583)	5.333 (4.667–5.667)	4.667 (4.000–5.000)	9.088	0.011
Hope	5.250 (4.500–5.870)	5.000 (5.000–5.500)	4.500 (4.000–5.500)	6.192	0.045

*P*_25_, *P*_50_, and *P*_75_ represent the lower quartile, median and upper quartile in the interquartile range.

As shown in Tables 6 and 7, there were significant differences among the three groups in the total score of psychological capital (*F* = 8.607, *p* = 0.000), self-efficacy (*H* = 6.377, *p* = 0.041), hope (*F* = 10.403, *p* = 0.000), resilience (*H* = 9.088, *p* = 0.011) and optimism (*H* = 6.192, *p* = 0.045). To further verify which two groups have significant differences, *post hoc* multiple comparisons were performed in this study. For the psychological capital and hope dimensions subject to a normal distribution, the Scheffe method was adopted for *post hoc* multiple comparisons (Wu, 2010; see Table 8). For the dimensions of self-efficacy, resilience, and optimism that did not obey the normal distribution, a Pairwise comparison between groups was also conducted, and a Calibration test based on the Bonferroni method was adopted (see Table 9).

**Table 8:** *Post hoc* multiple comparisons based on Scheffe method.

Variable	Grouping comparison		Mean difference	Significance
Psychological capital	Structured reading materials group	Mindfulness intervention group	0.011	0.997
		Control group	0.521	0.002
	Mindfulness intervention group	Control group	0.510	0.003
Hope	Structured reading materials group	Mindfulness intervention group	−0.002	0.999
		Control group	0.616	0.001
	Mindfulness intervention group	Control group	0.618	0.001

**Table 9:** Calibration test based on Bonferroni method.

Variable	Grouping comparison		Standard inspection statistics	Adjusted significance
Self-efficacy	Structured reading materials group	Mindfulness intervention group	0.165	1.000
		Control group	2.271	0.069
	Mindfulness intervention group	Control group	2.085	0.111
Resilience	Structured reading materials group	Mindfulness intervention group	−0.581	1.000
		Control group	2.301	0.064
	Mindfulness intervention group	Control group	2.832	0.014
Optimism	Structured reading materials group	Mindfulness intervention group	0.354	1.000
		Control group	2.315	0.062
	Mindfulness intervention group	Control group	1.940	0.157

As shown in Tables 8 and 9, in the overall psychological capital, the structured reading material group was significantly higher than that of the control group (*p* = 0.002), and the mindfulness intervention group was also significantly higher than that of the control group (*p* = 0.003). The overall level of psychological capital of the structured reading material group was slightly higher than that of the mindfulness intervention group, but there was no significant difference (*p* = 0.997). Comprehensive test results also showed that the structured reading material and mindfulness intervention had no significant difference in any dimension; H3 was non-supported.

### 4.5. Hypothesis test results

To sum up, the results of hypothesis testing are shown in Table 10. Only H1 was totally supposed.

**Table 10:** Hypothesis test results.

Hypothesis	Overall test	Different dimensions test			
		Self-efficacy	Hope	Resilience	Optimism
H1: Structured reading materials could improve the psychological capital of new venture entrepreneurs.	Supported	Supported	Supported	Supported	Supported
H2: Mindfulness intervention could improve the psychological capital of new venture entrepreneurs.	Supported	Supported	Supported	Supported	Non-supported
H3: The effect of structured reading materials on developing new venture entrepreneurs’ psychological capital is better than mindfulness intervention.	Non-supported	Non-supported	Non-supported	Non-supported	Non-supported

## 5. Discussion and conclusion

This study was implemented using an online experiment with control and experiment groups alongside pre-test and post-test. Psychological capital development methods were adopted based on the literature review and expert discussions. These methods fit new venture entrepreneurs’ characteristics and are practical and operational. Through online experiments, this study tested the effectiveness of the psychological capital development of new venture entrepreneurs under COVID-19. Furthermore, the effects of structured reading materials and mindfulness intervention were compared.

Conclusion 1: The psychological capital of new venture entrepreneurs under COVID-19 could be developed in specific ways. First, structured reading materials could effectively improve new venture entrepreneurs’ psychological capital and various dimensions. This is similar to the results of previous studies. A study took a non-teaching staff of a university in Iranian as objects and found that psychological capital was significantly improved based on reading therapy and could bring material and spiritual benefits to the organization (Papi et al., 2017). While learning the knowledge of psychological capital and experiencing the experiences of others, the subjects identified their weakness from an old and ineffective understanding of psychological capital. Obtained emotional support and immersive experience, reshaped individual awareness and inappropriate way of thinking, endowed themselves with positive energy in the new behavior pattern, and finally realized the improvement of psychological capital level. In general, structured reading materials were easy to use, low-cost, and suitable for large-scale use during COVID-19.

Secondly, mindfulness intervention could effectively improve the psychological capital, self-efficacy, hope, and resilience of new venture entrepreneurs, but the improvement of optimism was limited. Mindfulness intervention helped individuals to become aware of their current state, avoid being immersed in negative emotional experiences, and work with a more positive attitude (Good et al., 2016). Unlike previous studies, this study found no evidence that mindfulness training significantly improved individuals’ optimism levels. This might be because the initial level of optimism in the mindfulness intervention group was higher than in the other two groups. Because of the glass ceiling effect, the relatively high baseline optimism level did not lead to significant improvement. In addition, the effect of mindfulness intervention was not evenly distributed among the four dimensions due to the short duration of mindfulness training or the lack of essentials in the practice process, resulting in the difference in the improvement effect of different dimensions.

Conclusion 2: There was no statistically significant difference in the effects of different psychological capital development methods, but the intervention effect of structured material reading was more balanced, which could produce certain effects on all dimensions of psychological capital. In contrast, mindfulness intervention had no statistical significance on the improvement of the optimistic dimension. There were two reasons for the difference in effectiveness between groups. On the one hand, it was the degree of participant involvement. The participants with higher involvement actively cooperated, had higher recognition of the expected goal of the intervention, and were more likely to learn the intervention content, so the intervention effect was ideal. Structured reading materials were generally simple, easy to understand, informative, and interesting, and participants had a high degree of involvement in the intervention process. However, mindfulness intervention was not well known to the public, and participants’ acceptance and recognition of this method were not as good as that of structured reading materials. Moreover, mindfulness intervention’s core abilities and skills were professional, which was difficult for beginners to get familiar with and master quickly. They needed to invest more energy and practice more. Another aspect was the nature of intervention methods. Mindfulness training usually takes longer and requires several consecutive exercises to achieve significant results. The improvement effect of a short intervention might not be significant. A study found a significant positive correlation between practice time and mindfulness level through MBSR (Carmody and Baer, 2008). Structured reading materials were based on PCI, and the short-term intervention effects of the PCI model were supported in different situations and populations (Luthans et al., 2006), allowing the structured reading materials group to acquire psychological capital thinking and behavior patterns in a short period.

Structured reading materials intervention is a good way to develop psychological capital. We cannot define structured reading as a reading activity but as the combination of reading and reflecting the reading. One crucial element of structured reading is the dialog and the post-activities that help further co-create the story and build new meanings about the situation in a positive way. So, after delivering the reading materials, helping them to read, and interacting with them, we researchers were always online to improve the readability of the material and determine the reading effect. With the development of the internet, structured reading inventions could be written materials or computer programs or the listening/viewing of audio/videotapes chosen by convenience and for different purposes.

## 6. Limitations and future work

Firstly, due to the small sample size and the significant differences in age, education, and industry among participants, this study might not completely balance the demographic variables between different groups in the random allocation process. These differences might weaken the effectiveness of the results. In terms of sampling scope, this study was mainly concentrated in Hubei, China, and the differences in regional culture, entrepreneurial environment, and enterprise scale might impact the universality of the research conclusion. Future research could further test the validity and external validity of the findings of this study by recruiting more participants and expanding the sampling range.

Secondly, to a certain extent, online intervention delayed the timely exchange and feedback of information, and communication lags. In the intervention process, it was difficult to close the psychological distance between the subjects and the experimenters only through written communication, so they were willing to devote themselves to and cooperate with the experiment. This study attempted to weaken the inherent defects of online intervention by selecting development methods that match the online experiment, increasing the interest of the experiment by adding games like grabbing red-envelope play links and active interaction topics. Still, the effect remained to be further tested. In addition, there were some uncontrollable factors for online intervention to compromise the results, whether participants had completed the full training. Future research can adopt online and offline mixed intervention designs to build a harmonious team atmosphere through offline activities and improve the investment in online intervention.

Thirdly, this study was implemented at a particular moment during the pandemic of COVID-19. Whether the research conclusion could be extended to other specific and conventional situations remained to be tested.

Finally, this study only examined the immediate effect of short-term intervention and did not test the long-term development effect of structured reading materials and mindfulness intervention. Future research can further consider the continuous development effect of psychological capital interventions in different situations and include multi-time point tracking data to test the time effect of psychological capital development.

## Data availability statement

The raw data supporting the conclusions of this article will be made available by the authors, without undue reservation.

## Ethics statement

Ethical approval for this study and written informed consent from the study participants were not required in accordance with local legislation and national guidelines.

## Author contributions

NZ participated in data collection and analysis, critically revising the whole manuscript. MZ designed this work, supervised the study, and finally approved this version. SF drafted the manuscript, reviewed the literature, and participated in data interpretation. QX drafted the manuscript and participated in data collection and data analysis. TJ critically revised the whole manuscript. All authors contributed to the article and approved the submitted version.

## Conflict of interest

The authors declare that the research was conducted in the absence of any commercial or financial relationships that could be construed as a potential conflict of interest.

## Publisher’s note

All claims expressed in this article are solely those of the authors and do not necessarily represent those of their affiliated organizations, or those of the publisher, the editors and the reviewers. Any product that may be evaluated in this article, or claim that may be made by its manufacturer, is not guaranteed or endorsed by the publisher.

## Supplementary material

The Supplementary material for this article can be found online at: https://www.frontiersin.org/articles/10.3389/fpsyg.2022.963439/full#supplementary-material
