# Supplementary material for: Online experimental research on the psychological capital development of new venture entrepreneur under the COVID-19 pneumonia epidemic
Source: Front Psychol. 2023 Jan 11;13:963439. doi: 10.3389/fpsyg.2022.963439 (PMC9874676; doi:10.3389/fpsyg.2022.963439)
Supplement: Supplementary file 1 [file Data_Sheet_1.docx]

**Appendixes:**

| **Structured Reading Material** | |
| --- | --- |
| Subject of Materials | Overview of Content |
| 一、Psychological Capital Lead-In | This part includes two aspects: definition and effect of psychological capital appreciation (hereinafter referred to as PC). First, to introduce existing dimensions of PC and explain the meanings of four dimensions in popular language according to Luthans and other scientists' definitions. Second, to indicate the features of what individuals with high-level PCA shall contain by displaying positive functions of PC from individual and organizational levels based on the review of researches. |
| 二、Upgrade Strategies and Case Materials for Hope | Upgrade Strategies: set sensible and resilient goals; plan the path to the goal; define available resources. |
|  | Case: i. Field adventure experiment: In the experiment, subjects in three groups walked to a village ten kilometers away. The subjects in first group did not know the name of the village or the distance to the village either, only told them to go with a guide. Some of them started to complain when they went not far, and felt more depressed the further they went. The second group merely knew the name of and way to the village but there was no landmark on the way, only dependent on the experienced subjects estimating journey time and the remaining distance. Despite exhaustion, the group eventually held on to the end with the clue of half a three-fourths course. The third group not only knew the name, and distance but also could see a milestone at intervals of one kilometer. With a definite target and high spirits, the group arrived at the destination in a short time. ii. A Harvard study: a survey by Harvard university targeted at young employees found: 27% of employees had no goals; 60% of them had goals but vague; 10% had clear but short-term goals; merely 3% of employees had clear and long-term goals. Tracking studies through 25 years indicated that their achievements are related to whether or not they have goals or whether the goals are clear, and that the employees with clear and long-term goals are somewhat the top dog of all walks of society. |
| 三、 Upgrade Strategies and Case Materials for Optimistic | Upgrade Strategies: embrace the past; cherish the present; seek the future; develop an optimistic explanatory style. |
|  | Case: i. In the year-end KPI assessment, the result of a new employee called Zhang Li was not desirable. He believed that he was born with such a hard task while his boss thought that his maladaptation led to a bad mood and attitude, and in the end, made a bad work performance. After discussion and comparison with his colleagues, he attributed his low KPI to a bad mood and attitude, then adjusted himself to the new job quickly and eventually got a good result. ii. Former British prime minister Lloyd George got used to closing the door behind him. One day, his friend doubted about this behavior, while Lloyd George explained: “when you close the door, you leave your past behind you whether the past is a brilliant accomplishment or a remorseful error, and then you can restart.” |
| 四、Upgrade Strategies and Case Materials for Self-efficacy | Upgrade Strategies: proficient grasp, experience success; alternative learning/imitation; social persuasion and positive feedback; psychological and physiological awakening and health. |
|  | Case: A Du, out of poor birth, made a living for his family at the young age of 15 years old. He first entered a garage, then a computer company, and at last came to a construction site where he had worked for seven years. The dull and seemingly hopeless life on the construction site, however, did not make him give up his music dream. Out of his love of music, he practiced singing all the time at the interval of break in a vacant land, and held on to the end though people around could not understand. His love and practice paid off: he stood out in an audition contest by accident and set foot in the circle of singers. Later, A Du signed with Sony, released albums in succession and won many music awards. His experience proved that in the face of adversity, only holding on straight to the end can make success. |
| 五、Upgrade Strategies and Case Materials for Resilience | Upgrade Strategies: concern on resilience capital; concern on hazard factors; focus on process. |
|  | Case: Helen Keller was attacked by acute encephalitis at 19 months old, resulting in loss of hearing and sight. However, she entered Cambridge girls' school with good performance, which was so difficult for a disabled person. When she was compiling a book *Teacher*, she was confronted with a fire, which destroyed her 20 years of effort. She otherwise did not give up and was much more resolved to finish the compiling of the book. Later, she joined the American Foundation for the blind. As the counselor connecting home and overseas, she traveled to many countries and strived to build schools for the blind. She often visited patients in hospitals and shared her individual experience and the will to live. |
| 六、Question Check | 1) What kind of concept of psychology closely related to our life and work is discussed in the essay? |
|  | 2) What are the four positive features included in psychological capital? |
|  | 3) Which upgrade strategy of psychological feature does “embrace the past， cherish the present, seek the future” belong to? |
|  | 4) Which upgrade strategy of “correctly assess oneself, fully recognize individual capitals one has owned, and hold on to, recover quickly and adopt a roundabout way to make success when meeting with problems and difficulties ” is? |
|  | 5) Which upgrade strategy of “set objectives related to jobs, make plans to realize and confirm obstacles to overcome” is ? |

***Data Source:***

*[1] Luthans, F., Youssef-Morgan, C M., and Avolio, B J. (2008). Psychological Capital: Developing The Human Competitive Edge, translated by Li C; China Light Industry Press: China, Beijing.*

*[2] Wang, D. (2012). A comparative study on the effectiveness of different intervention methods of psychological capital. Master degree. Beijing Normal University, China (in Chinese).*

| **Mindfulness Intervention** | |
| --- | --- |
| Subject of Materials | Overview of Content |
| 一、Mindfulness Lead-in | This part includes the following contents: to introduce Kabat-Zinn's definition of mindfulness and the origin of mindfulness; to learn the benefits of mindfulness practice; to analyze the theory of mindfulness psychological therapy; to identify the key points of body scan and breath perception. |
| 二、Mindfulness Practice | 15-minute breath perception; 15-minute body scan. |
| 三、Question Check | 1) What kinds of mindfulness practices do occur in the experiment? |
|  | 2) What is the key point of body scan? |
|  | 3) What is the key point of breath perception? |
|  | 4) How long do you do mindfulness practice? |

***Data source:*** *it is organized according to relevant literature.*
